# ATM Expression Is Elevated in Established Radiation-Resistant Breast Cancer Cells and Improves DNA Repair Efficiency

**DOI:** 10.7150/ijbs.41246

**Published:** 2020-02-04

**Authors:** Lei Bian, Yiling Meng, Meichao Zhang, Zhuying Guo, Furao Liu, Weiwen Zhang, Xue Ke, Yuxuan Su, Meng Wang, Yuan Yao, Lizhong Wu, Dong Li

**Affiliations:** 1 Department of Radiation Oncology, Shanghai Ninth People's Hospital, Shanghai Jiaotong University School of Medicine, Shanghai, China; 2 Department of Clinical Laboratory, Shanghai Ninth People's Hospital, Shanghai Jiaotong University School of Medicine, Shanghai, China; 3 Department of Radiology, Shanghai Ninth People's Hospital, Shanghai Jiaotong University School of Medicine, Shanghai, China

**Keywords:** breast cancer, radiation, DNA damage repair, ATM

## Abstract

Repair of damaged DNA induced by radiation plays an important role in the development of radioresistance, which greatly restricts patients' benefit from radiotherapy. However, the relation between radioresistance development and DNA double-strand break repair pathways (mainly non-homologous end joining and homologous recombination) and how these pathways contribute to radioresistance are unclear. Here, we established a radioresistant breast cancer cell line by repeated ionizing radiation and studied the alteration in DNA repair capacity. Compared with parental sham-treated cells, radioresistant breast cancer cells present elevated radioresistance, enhanced malignancy, increased expression of Ataxia-telangiectasia mutated (ATM), and increased DNA damage repair efficiency, as reflected by accelerated γ-H2AX kinetic. These defects can be reversed by ATM inhibition or ATM knockdown, indicating a potential link between ATM, DNA repair pathway and radiosensitivity. We propose that cancer cells develop elevated radioresistance through enhanced DNA damage repair efficiency mediated by increased ATM expression. Our work might provide a new evidence supporting the potential of ATM as a potential target of cancer therapy.

## Introduction

Radiation therapy is efficacious, cost-effective for many solid tumors. About 50% of cancer patients receive radiation therapy and 40% of cancer cure is believed to be related to radiation treatment 1, 2. For example, along with chemotherapy, radiation therapy brings in a benefit of an absolute 10% reduction in the risk of disease-related death for breast cancer patients 3, 4. However, an adaptive response of cancer cells to radiation leads to the development of radiation resistance which ultimately leads to tumor recurrence and metastasis. As a result, the application of radiation therapy is still limited 5.

The anticancer efficacy of radiation is mainly due to its ionization effect. It produces oxygen free radicals and the latter causes a broad spectrum of DNA lesions 6. Amongst lesions induced by ionizing radiation, double-strand breaks (DSBs) are the most lethal type, which could lead to cell apoptosis or necrosis 7. To repair DSBs, two main DNA damage repair (DDR) pathways, homologous recombination (HR) and non-homologous end joining (NHEJ), are activated. These two main DSBs repair pathways play an indispensable role in initiating and processing of cancer. Patients harboring deficiencies affecting HR or NHEJ often present increased susceptibility towards cancer 8, 9. Nonetheless, alterations in DSB repair pathways play an important role in the development of radioresistance 10.

H2AX is a key component in DNA repair, which is rapidly phosphorylated on a serine four residues from the carboxyl terminus and forms γ-H2AX at nascent DSB sites. During a short time after DSB formation, γ-H2AX accumulates around the breaks and forms foci that could be detected 11. γ-H2AX correlates well with DSBs formed. When DSBs are repaired, γ-H2AX disappears. Thus, the kinetics of γ-H2AX is often used to monitor DSBs repair efficiency 12. NHEJ is the fast DSB repair pathway, which mainly contributes to the repair of DSBs in 2 hours after DSB formation 13. Furthermore, NHEJ is believed to be the preferred pathway for the repair of IR-induced DSBs 14. Some research indicated that it is HR repair efficiency, not NHEJ, that is significantly elevated in breast cancer cells compared with normal mammalian cells 15. However, little is done to compare HR and NHEJ efficiency in relatively radio-resistant and radio-sensitive cells from the same origin, which might better reflect how the adaptation of radiation is developed in cancer cells.

Ataxia-telangiectasia mutated (ATM) encodes a serine/threonine kinase that belongs to the phosphatidyl inositol 3-kinase (PI3K) like protein kinases (PIKKs) family 16. It functions as an important DSBs sensor 17. In the HR pathway, many essential effectors are phosphorylated and activated by ATM. ATM also functions in the NHEJ pathway through phosphorylating kinase DNA-PKcs and nuclease Artemis 18. Several studies showed that there was some associations between radiosensitivity and ATM expression levels 19-21. Upregulation of ATM protein and its activation are reported to be correlated with enhanced radioresistance 22-24. A combination of chemotherapy and ATM inhibitor is a promising new cancer treatment under trials nowadays 25.

In our present work, we established a relatively radio-resistant breast cancer cell line and found that γ-H2AX kinetic was significantly altered in the cells, indicating an elevated DNA damage repair response. Further experiments suggested that this might be mediated by ATM, a molecule vital for HR and NHEJ. Thus, the prediction of instinct sensitivity towards ionization therapy based on detecting DSB repair efficiency of cancer cells or monitoring ATM expression might be a promising new strategy for cancer treatment.

## Methods

### Antibodies and reagents

The following reagents were used: I-SceI enzyme (NEW ENGLAND Biolabs, #R0694S). TIANgel Midi Purification Kit (TIANGEN, #DP209-02), Hieff Trans™ Liposomal Transfection Reagent (Yeasen, #40802ES08), Apoptosis Analysis Kit (Yeasen, #40301ES50), CCK-8 kit (DOJINDO, #CK04-3000T). Paclitaxel (#HY-B0015), Doxorubicin (#HY-15142A) and CP-466722(HY-11002) were purchased from MedChemExpress and dissolved in dimethyl sulfoxide (DMSO) for stocking solutions and stored according to manufacturer's instructions.

Antibodies used in western blotting and immunofluorescence are listed in Table. S1.

### Cell cultures and the establishment of radioresistant cell line

Breast cancer cell line MDA-MB231 was purchased from ATCC and cultured following the protocol: cultured with the medium of DMEM medium (Gibco) supplemented with 10% fetal bovine serum (FBS) and maintained in a humidified atmosphere at 37 ˚C with 5% CO_2_.

For the establishment of the radioresistant cell line, parental MDA-MB-231 cells were divided into two groups, MDA-MB-231-PB and MDA-MB-231-PR. 1x10^6^ cells were seeded in 10cm culture disks on day 1. On day 3 or 4, when cells attained 60-70% confluency, MDA-MB-231-PR cells received 3Gray (Gy) of X-ray (Elekta) irradiation at a dose rate of 1.43Gy per minute. Cells were trypsinized, counted and passed into new dishes when cell confluency reached approximately 90%. Medium was changed every other day. The irradiation was performed 20 times for a total dose of 60Gy over 4 months. MDA-MB-231-PB cells were treated under the same condition without the irradiation.

### Clonogenic survival and linear-quadratic-model

Proper numbers of cells were plated in 6-wells cell culture plates in triplicate and allowed 24 hours for attachment. After 24 hours, cells were irradiated with 0-6Gy of X-ray at a dose rate of 1.43Gy per minute and then incubated 10 to 14 days for colony formation. Colonies were fixed with 4% paraformaldehyde and stained with crystal violet. Colonies of >50 cells were counted as clonogenic survivors. Surviving fraction (SF) after a certain dose was calculated. SF(d)= [number of clonogenic survivor (d)] / [number of clonogenic survivors (0)] while d indicates radiation dose and 0 means when dose is 0. Linear-quadratic -model is described below:

 S= e^-d (α + βd)^

Here, e represents the natural logarithm, with S is the surviving fraction after a certain dose of ionizing radiation.

### Western blotting

Western blotting was performed according to a standard protocol. Briefly, cells were lysed in lysis buffer 26 and were analyzed by immunoblotting after SDS-PAGE. Proteins were visualized by ECL according to the manufacturer's instructions (Millipore).

### Immunofluorescence staining

Cells were seeded on coverslips placed in 12-well plates. 24 hours later, cells were irradiated as indicated. Cells were then fixed, permeabilized and blocked at the indicated time. Anti-Phosphor-Histone H2A.X(Ser139) and Donkey anti-mouse IgG Alexa Fluor conjugate were used for staining. Dapi was used for nuclear staining. Pictures were taken using a Nikon A1 confocal microscope.

### Homologous recombination and Non-Homologous End Joining repair efficiency

Plasmids for the detection of HR and NHEJ were generously provided by Dr. Vera Gorbunova (University of Rocherster, New York). Experiments were described previously 15. In brief, the reporter substrate contains a GFP sequence with an inserted intron, which does not affect GFP expression. The intron is interrupted by an adenoviral exon, which is flanked by restriction sites of I-*Sce*I for DSBs. Upon treatment with I-*Sce*I, these restriction sites are recognized and digested, thus the adenoviral exon is cut out, DSBs are left which could be repaired by either HR or NHEJ. When the DSBs are repaired, the intron is ligated and the GFP expression is restored.

NHEJ-Incompatible (NHEJ-I), NHEJ-Compatible (NHEJ-C), and HR plasmids were linearized using I-SceI enzyme. 1ug linearized plasmids and 1ug DsRed plasmid (used as transfection efficiency control) were co-transfected into cells. 72 hours later, cells were trypsinized and washed with cold phosphate-buffered saline (PBS), and subjected to flow cytometry analysis. The repair efficiency was calculated as the number of cells with green fluorescence/the number of cells with red fluorescence.

### siRNA knockdown

Small interfering RNA (siRNA) transfection was carried out using Lipofectamine 3000 (Thermo Fisher Scientific). siRNA nucleotides against negative control (NC) and ATM were obtained from GenePharma. The sequence of siRNA oligonucleotides against ATM was 5'-GCU UGA GGC UGA UCC UUA UTT-3' (Si-1), and 5'-GCA AAG CCC UAG UAA CAU ATT-3'(Si-2).

### Flow cytometry

Flow cytometry was done using FACS Canto™ flow cytometer. Cell cycle analysis was performed using the Cell Cycle and Apoptosis Analysis Kit (Yeasen, #40301ES50) according to the manufacturer's instructions. Apoptosis analysis was performed using Annexin V-FITC/PI kit (Yeasen, #40302ES20). Cells were subjected to radiation exposure or sham-treated. After the indicated time of incubation, cells were trypsinized and washed with PBS, then dyed with Annexin V-FITC/PI reagents before the flow cytometry analysis.

### Statistical analysis

Results were expressed as mean
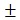
SD. Data were analyzed by using student's test. *P* values <0.05 were considered significant.

## Results

### Establishment of radioresistant breast cancer cell line

Parental MDA-MB-231 cells were divided into two subsets. One received 20 times of fractioned irradiation with a total dose of 60Gy and designated as MDA-MB231-PR (MD-PR) cells. Another group was treated under the same condition but received no irradiation and named as MDA-MB231-PB (MD-PB) cells.

**Figure 1:**
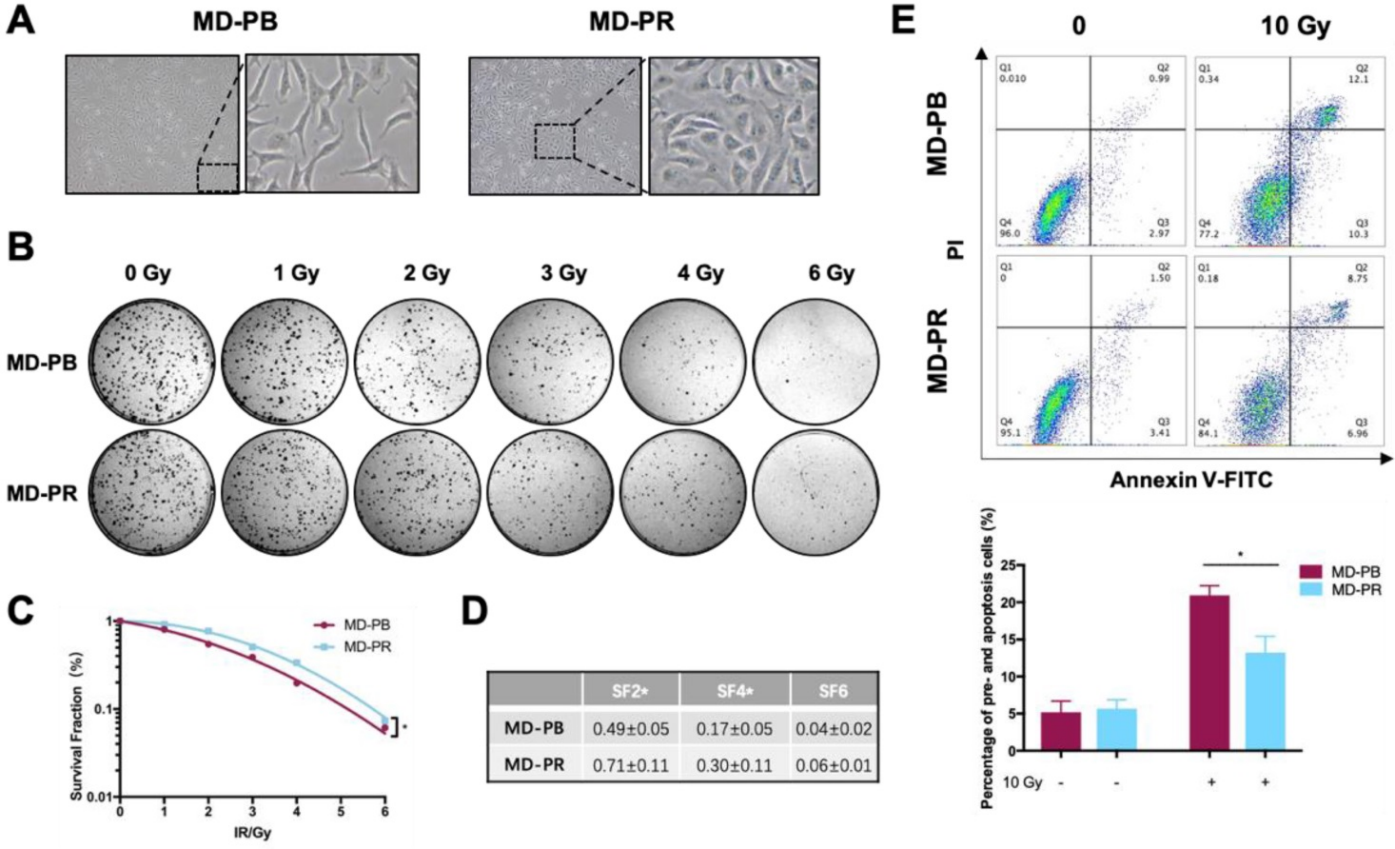
** MD-PR cells present enhanced radioresistance compared with MD-PB. (A)** Morphology changes of MD-PB and MD-PR under the microscope of 100X magnification, with representative cells zoomed in on the right. **(B-D)** MD-PB and MD-PR cells were seeded in a 6-well plate in triplicate. 24-hours later, cells were subjected to 0-6 Gy of X-ray radiation (Elekta, 1.43 Gy/min). After 10-14 days of incubation, formed clones were fixed, stained and counted. Surviving fraction was calculated and fitted into the linear-quadratic model as described in the Materials and Methods **(C).** A representative image of three independent experiments was showed **(B)**. Surviving fraction at certain doses as indicated in **(D)**. **(E)** MD-PB and MD-PR cells were exposed to 0 or 10 Gy of X-ray. 48 hours later, cells were collected, stained with PI and Annexin V-FITC dye and subjected to flow cytometry analysis. Annexin-V-positive cells (Q3) were counted as pre-apoptotic cells and PI-positive, Annexin-V-positive cells (Q2) were apoptotic. Percentage of apoptotic cells equals the sum of Q2 and Q3. Top panel: one representative result of apoptosis analysis. Bottom panel: the statistic results of 3 separate experiments. (* *p*<0.05)

The morphology of MD-PR cells was obviously different from that of MD-PB cells under microscopy (Fig. 1A). MD-PR cells had a much more stretched and flatter appearance compared with the latter. We then examined whether the abilities of migration and invasion were changed in MD-PR cells. Results showed that MD-PR cells had increased migration and invasion capacities compared with MD-PB cells (Fig. S1A, B). Increased expression of mesenchymal markers (N-cadherin, Snail, Slug and beta-catenin) and decreased expression of epithelial marker E-cadherin in MD-PR were also detected (Fig. S1C). These results suggested that enhanced malignancy was induced in MD-PR cells 27.

The radiosensitivity of MD-PB cells and MD-PR cells was compared. Various parameters reflected that the MD-PR cell line had a higher survival rate compared with MD-PB cells (Fig. 1B, C, D). The surviving fraction at 2 Gy (SF2) is a commonly used parameter for measurement of radiosensitivity *in vitro* 28, 29. SF2 was significantly increased in MD-PR cells compared with MD-PB cells (0.71±0.11 vs 0.49±0.05,* p* = 0.04) (Fig. 1D) along with significant changes in SF4. Enhanced radioresistance in MD-PR cells was furtuher evidenced by apoptotic assays. Results of apoptotic assays showed that, after a large dose of irradiation, the proportion of pro-apoptotic (Annexin V-FITC positive) and apoptotic (Annexin V-FITC positive, PI positive) cells was significantly reduced in MD-PR cells compared with MD-PB cells (13.22±2.17 vs 20.92±1.33,* p*=0.01, Fig. 1E). The above results showed that MB-PR cells were more radioresistant compared with MD-PB cells.

At the same time, we tested whether repeated irradiation could change the cell proliferation rate and cell cycle distribution. The results showed that there was no significant difference between MD-PR cells and MD-PB cells in these aspects (Fig. S1D, E).

### Altered γ-H2AX kinetic in radioresistant MD-PR cells

To find the possible mechanism explaining reduced apoptosis ratio in MD-PR cell line after irradiation, DSB repair efficiency was evaluated in MD-PB and MD-PR cells by detecting Phosphor-Histone H2A.X (Ser139) (γ-H2AX), which was a widely adopted marker for the detection of DSBs 11.

**Figure 2:**
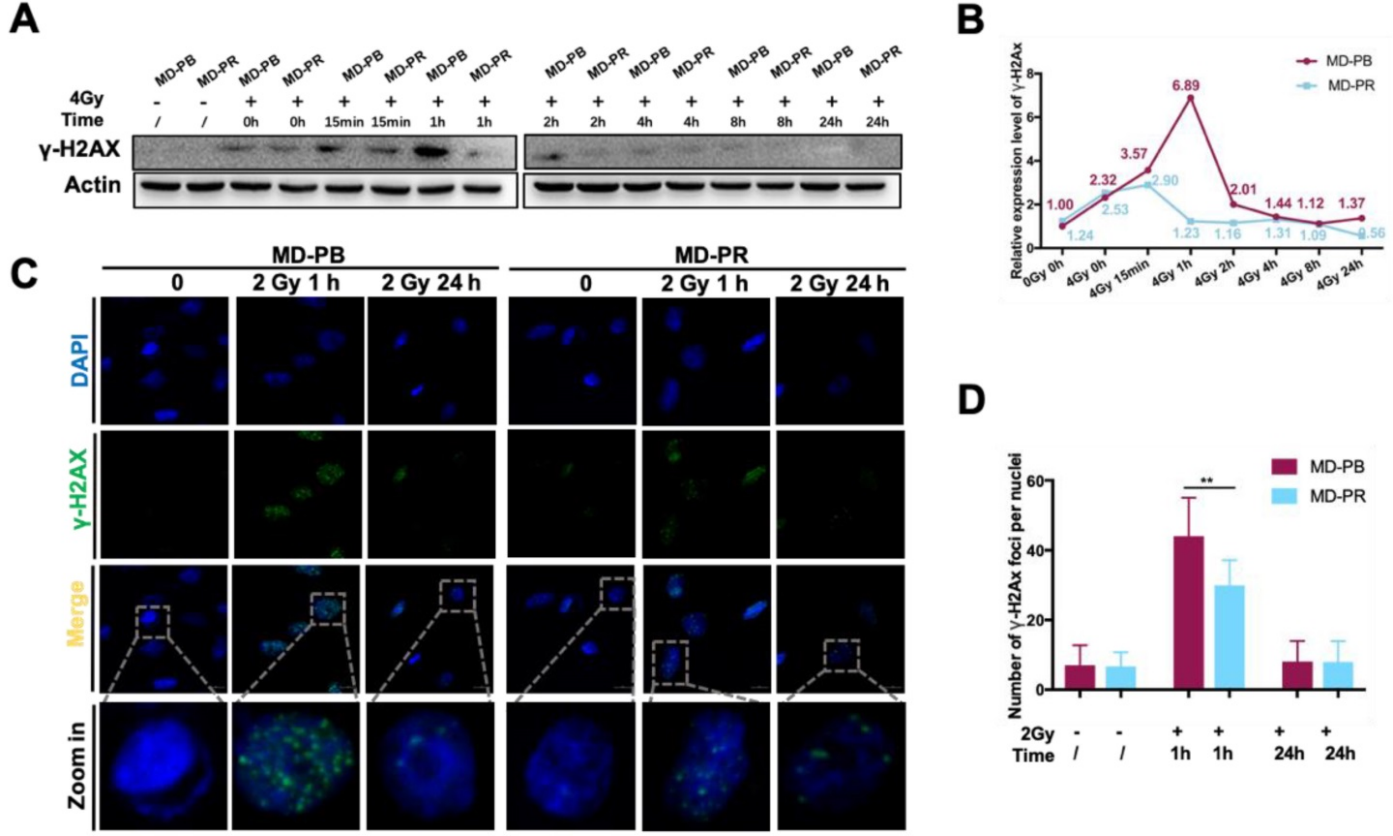
** Altered γ-H2AX kinetic in MD-PR cells. (A)** Cells were exposed to 0 or 4 Gy of irradiation and lysed at the indicated times for analysis of γ-H2AX with Western-blotting. Actin was used as loading control. **(B)** Quantitative analysis of the blots. Plotted is the relative increase of γ-H2AX compared with MD-PB at 0 Gy as a function of post-irradiation incubation time, with statistics on the bottom. **(C)** Immunofluorescence of γ-H2AX in cells treated with or without 2 Gy of radiation. Samples were collected at indicated time. γ-H2AX was stained with green and nucleus with blue. Pictures were taken under 1600x magnification, with representative cells zoomed in at the bottom. Foci formed in nuclei was counted in at least 5 random fields and the statistical results were shown in D. (** *p*<0.001)

Western blotting experiments evidenced that kinetics of γ-H2AX varied between MD-PR and M-PB cells (Fig. 2A, B). γ-H2AX expression peaked at 15 minutes after irradiation and decreased to normal level at about 2-hour post-irradiation in MD-PR cells, while it attained a peak at 1-hour and decreased to normal level at 2-hour in MD-PB cells (Fig. 2B). The two patterns of γ-H2AX kinetics suggested that DSBs were repaired faster in MD-PR cells. Consistent with results of western blotting, the number of γ-H2AX foci per nuclei enumerated at 1-hour post-irradiation in MD-PR cells (30±7 foci per nuclei) was significantly lower than that in MD-PB (44±11 foci per nuclei) (Fig. 2C, D).

These results suggested an enhanced DSB repair response in MD-PR cells, which may explain the increased survival fraction in this cell line after different dose of irradiation.

### ATM was up-regulated in radioresistant MD-PR cells

Patterns of kinetic of γ-H2AX mainly differ within 2 hours after irradiation, suggesting that the NHEJ pathway, the fast DSB repair pathway 13, might be affected during the process of radioresistance development in MD-PR cells. For verification, reporter cassettes were used to detect the repair efficiency of HR and NHEJ 30.

**Figure 3:**
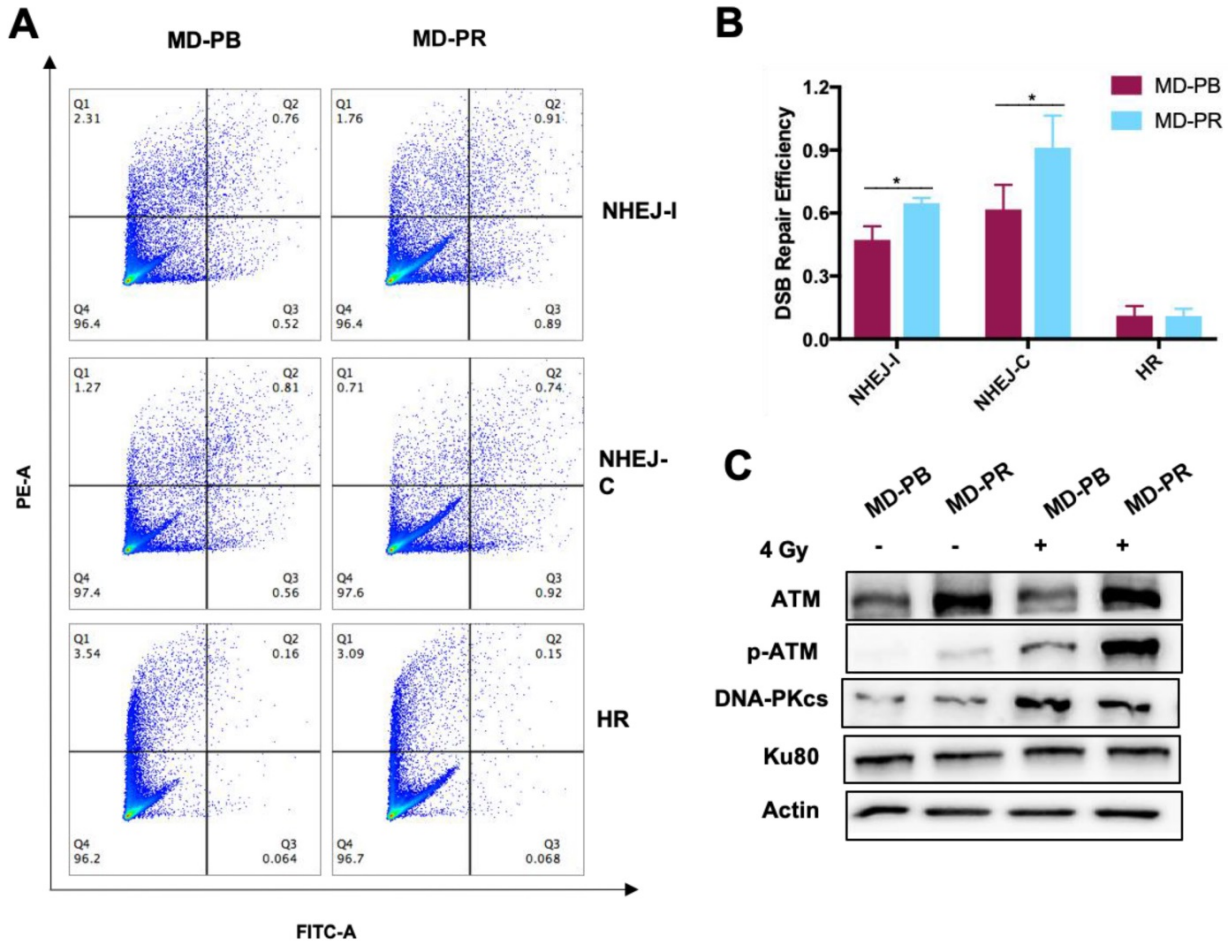
** Upregulated DDR efficiency and ATM expression level in MD-PR cells. (A-B)** Flow cytometry result of HR and NHEJ efficiency in MD-PR and MD-PB cells. NHEJ with compatible (NHEJ-C) and incompatible (NHEJ-I) ends and HR plasmids were linearized by I-SceI enzyme and transfected into cells with DsRed plasmid as a control. 72 hours later, cells were collected for flow cytometry analysis. The repair efficiency was calculated as cells with green fluorescence (Q2+Q3) / cells with red fluorescence (Q1+Q2). One representative result of flow cytometry analysis was shown in **(A)**. Statistical results of at least three independent experiments were shown in **(B)**. **(C)** Western blotting of NHEJ related markers. Cells were subjected to 4 Gy of radiation or sham-treated. Protein samples were collected for the test. (* *p*<0.05)

Firstly, we measured the HR or NHEJ efficiency in MD-PR and MD-PB cells. Results showed that the efficiency of NHEJ pathways was much higher than the efficiency of HR in both cell lines (Fig. 3A, B), which was in accordance with other researchers' results 30. Secondly, we investigated the efficiency of each DSB repair response pathway in MD-PR and MD-PB cells respectively. No obvious change was observed in HR efficiency in MD-PR cells compared with MD-PB cells (Fig. 3A, B). However, the repair efficiency for NHEJ-I in MD-PB cells was 0.47±0.06 and it increased to 0.65±0.02 in MD-PR cells, while that for NHEJ-C ascended from 0.62±0.12 to 0.91±0.15, both with significant difference (*p*< 0.05) (Fig. 3A, B). These results indicated that it is NHEJ, not HR, that is intensely activated in the radioresistant MD-PR cells.

NHEJ is an intricate pathway and many proteins, such as ATM, KU80 and DNA-PKcs, are involved 18, 31. Expression levels of these key proteins were detected (Fig. 3C) and ATM presented increased level in MD-PR cells. p-ATM (ser1981) is the activated form of ATM, whose existence is scarce without the trigger of DSBs. In both cell lines, the expression level of p-ATM increased dramatically after radiation (Fig. 3C). Consistent with elevated expression of ATM, p-ATM in MD-PR cells was much higher than that in MD-PB cells. No obvious difference in DNA-PKcs and KU80 between these two cell lines was observed (Fig. 3C).

Considering ATM as an important modulator of DDR efficiency and its impact on radiosensitivity 17, 19-21, we next studied the role of incresed expression of ATM in radioresistance development.

### ATM mediated the alteration of γ-H2AX kinetic and radiosensitivity in MD-PR cells

To figure out the role of ATM in radioresistance development, we used ATM specific inhibitor to reveal the impact of ATM on elevated radioresistance. CP466722 is an ATM specific inhibitor. The transient application of this compound is proved effective in inhibition of cellular ATM kinase activity, resulting in increased cell sensitivity towards radiation 21.

**Figure 4:**
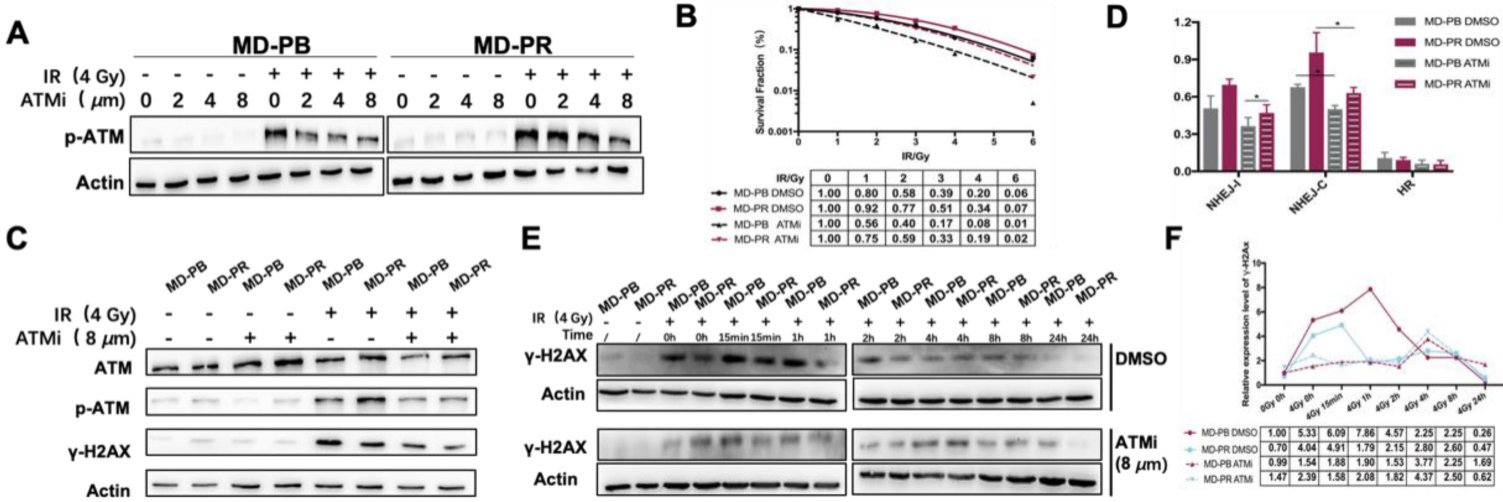
** ATM inhibition altered the γ-H2AX kinetic and radiosensitivity in MD-PR cells. (A)** Western blotting of p-ATM. Cells were incubated with the indicated concentration of ATMi (CP-466722) for 2 hours, and followed by treatment with or without 4 Gy of X-ray. Protein samples were collected 2 hours after irradiation and subjected to analysis.** (B)** Representative result of LQM of MD-PR and MD-PB cells with or without ATMi. Cells were incubated with DMSO or 8 *μ*M ATMi for 2 hours, and followed by exposing to radiation. 3 hours later, medium was completely changed. Surviving fraction was calculated as before and fitted into LQM with statistics shown at the bottom of the panel. **(C)** Cells were treated as indicated and lysed for western blotting. Samples were collected 1-hour post-irradiation. **(D)** NHEJ-I, NHEJ-C and HR repair efficiency in cells with DMSO or ATMi. Linearized plasmids and DsRed plasmid were cotransfected. 6 hours later, ATMi or DMSO was added. 72 hours after transfection, flow cytometry analysis was performed. **(E)** Kinetic of γ-H2AX was performed as described elsewhere. Cells were treated with ATMi or DMSO 2 hours before irradiation with the medium completely changed 3 hours post-irradiation. **(F)** Quantitative analysis of the blots. Plotted is the relative increase of γ-H2AX compared with MD-PB at 0 Gy with the treatment of DMSO as a function of post-irradiation incubation time with statistics shown at the bottom.

Our experiments showed that CP466722 at the concentration of 8 *μ*M was sufficient to inhibit the activation of ATM (Fig. 4A). So 8 *μ*M CP466722 would be used in the following experiments. Plating efficiency was similar with or without CP466722 treatment, indicating this compound had no significant effect on cell plating or cell viability (Fig. S2A). However, the presence of CP466722 did sensitize both MD-PB and MD-PR cells towards radiation, reflected by the surviving fraction. Especially, the inhibition of ATM kinase activity, even transient, re-sensitized MD-PR cells to ionizing radiation to a level similar to that of MD-PB with DMSO (Fig. 4B). Together, these results indicate that increased expression of ATM is correlated with enhanced radioresistance in MD-PR cells.

To evidence the impact of CP422766 on ATM, ATM and p-ATM were tested at the same time (Fig. 4C). No changes were observed for DNA-PKCs since CP466722 is ATM-specific (Data not shown). Since ATM is the main kinase responsible for phosphorylation of γ-H2AX 32, the application of CP422766 decreased γ-H2AX level in MD-PR and MD-PB cells after irradiation compared with the control group (Fig. 4C, E, F). Besides, ATM inhibition eliminated γ-H2AX kinetic differences in MD-PR and MD-PB cells (Fig. 4E, F). In both cell lines, γ-H2AX expression slightly increased and attained a peak at around 4-hour post-irradiation.

Since DSBs repair is mainly conducted by NHEJ in early time 33, We further checked DSB repair efficiency in MD-PB and MD-PR cells with or without CP422766. CP466722 significantly decreased NHEJ-C efficiency in both cell lines, and eliminated the difference of NHEJ-C between two cell lines. (Fig. 4D, Fig. S2B).

**Figure 5:**
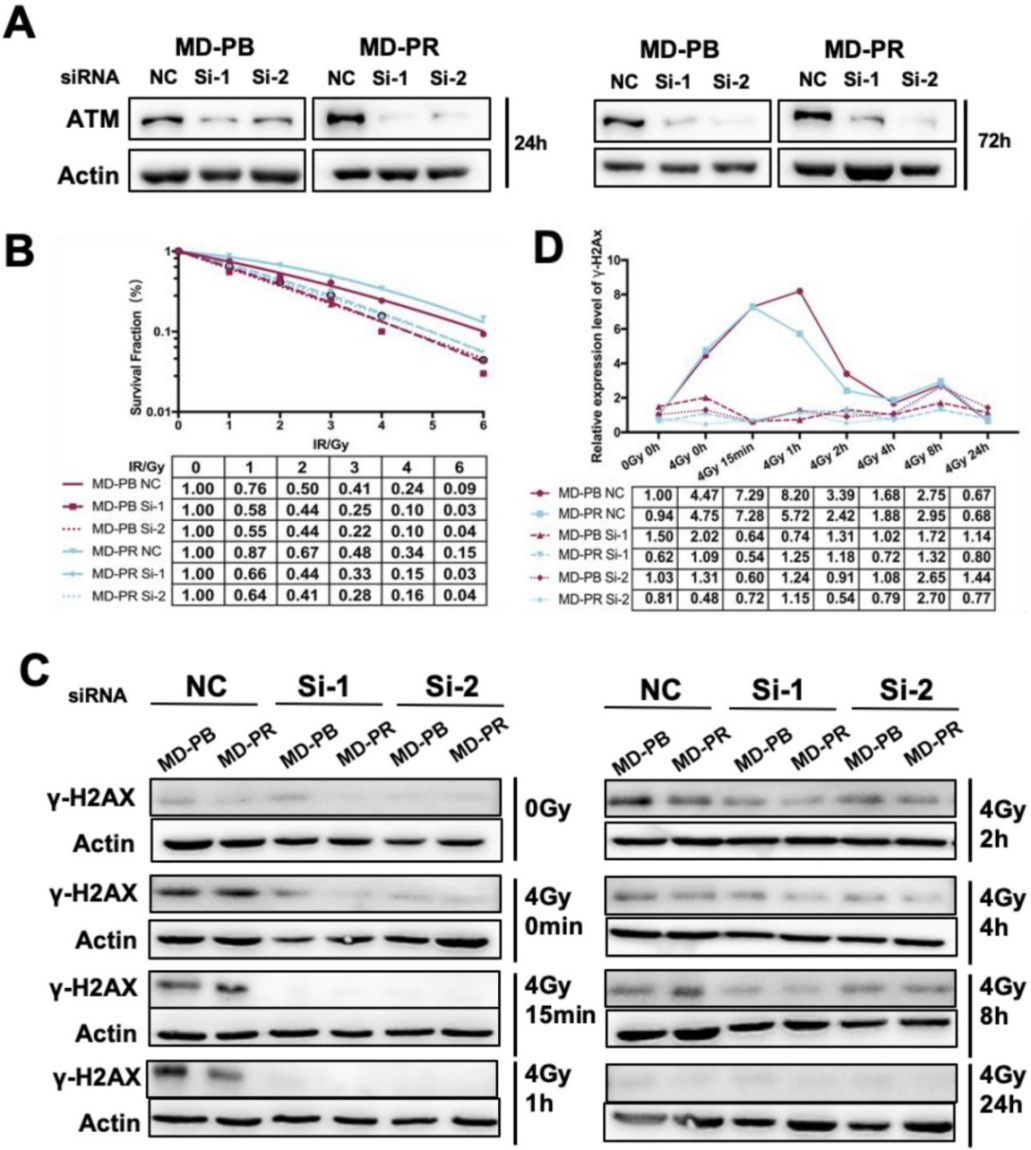
** ATM Knock-down sensitized cancer cells to irradiation and abrogated γ-H2AX expression. (A)** siRNA against ATM effectively reduced the expression of ATM at up to 72 hours after transfection. Logarithmically growing cells were transfected with siRNA and negative control oligonucleotides. Cells were lysed for ATM analysis at indicated time post-transfection. **(B)** ATM knock-down sensitized both cell lines to irradiation. Cells were transfected with siRNA or NC as described in Materials and Methods. 24 hours later, cells were seeded in 6-well plate in triplicate and allowed 24-hour for attachment before receiving 0-6 Gy irradiation. Fitting of linear-quadratic model was described in preceding part. **(C)** ATM knock-down abrogated phosphorylation of H2AX in siRNA treated cells. Cells were transfected with siRNA and reseeded 24 hours later, and allowed another for attachment before subjected to 0 or 4 Gy of X-ray. Cell lysates were collected at indicated time post-irradiation and used for the analysis of γ-H2AX. Actin was used as loading control. **(D)** Quantitative analysis of the blots. Plotted is the relative increase of γ-H2AX compared with MD-PB at 0 Gy as a function of post-irradiation incubation time with statistics shown at the bottom.

The high selectivity of CP422766 toward ATM has been documented 21. Nevertheless, to further confirm the specificity of CP422766, we exploited RNAi-induced knockdown to deplete ATM in MD-PB and MD-PR cells. Si-1 and Si-2 of ATM successfully interfered with the expression of ATM within 72 hours after transfection (Fig. 5A). In keeping with the results of ATM inhibitor, siRNA against ATM increased radiosensitivity in both cell lines and narrowed the gap of radioresistance between MD-PR and MD-PB (Fig. 5B). Apoptosis analysis after lethal irradiation further supported this result (Fig. S2C). The kinetics of γ-H2AX revealed that after ATM knock-down using siRNA, γ-H2AX was hardly detected in early time after irradiation with a slight peak at around 8-hour (Fig. 5C, D). These results furthur confirmed that altered γ-H2AX kinetic and radiorensiticity in MD-PR cells were mediated by elevated expression of ATM.

## Discussion

Radiation is one of the most effective therapeutic methods for cancer treatment. However, due to the intrinsic and therapy-induced radioresistance, it remains a conservative choice. In the process of radioresistance development, a pleiotropic of mechanisms are involved 10. Among them, DDR is one of great importance. Clarifying the mechanism underlying changed DDR in radioresistant cancer cells would provide a new insight into the radiosensitization of cancer cells.

To observe alterations in DDR caused by radiotherapy, we established one radioresistant cell line through repeated X-ray radiation and one cell line as control. These two cell lines have the same origin but present different radiosensitivities (Fig. 1). Significant enhancement in DSB repair efficiency, as reflected by accelerated kinetic of γ-H2AX, was observed in radioresistant cell line MD-PR (Fig. 2A-D). Results from the reporter assays (Fig.3B) suggest that the alteration in γ-H2AX kinetics may be caused by changes in NHEJ efficiency that are responsible for the repair of most DSBs during the early time after irradiation 33. Analysis of NHEJ related molecules found that ATM expression was increased in MD-PR cells (Fig.3C). Furthur experiments using ATM-specific inhibitor and siRNAs against ATM confirmed the relation between increased ATM expression, altered γ-H2AX kinetic and enhanced radioresistance. Also, MD-PR cells were more resistant to Doxorubicin (Fig. S1F) and Paclitaxel (Fig. S1G), two common chemotherapeutics for breast cancer treatment. Doxorubicin exerts its anti-cancer effect through inducing DNA damage 34. This further evidenced the enhanced DDR response in MD-PR.

**Figure 6:**
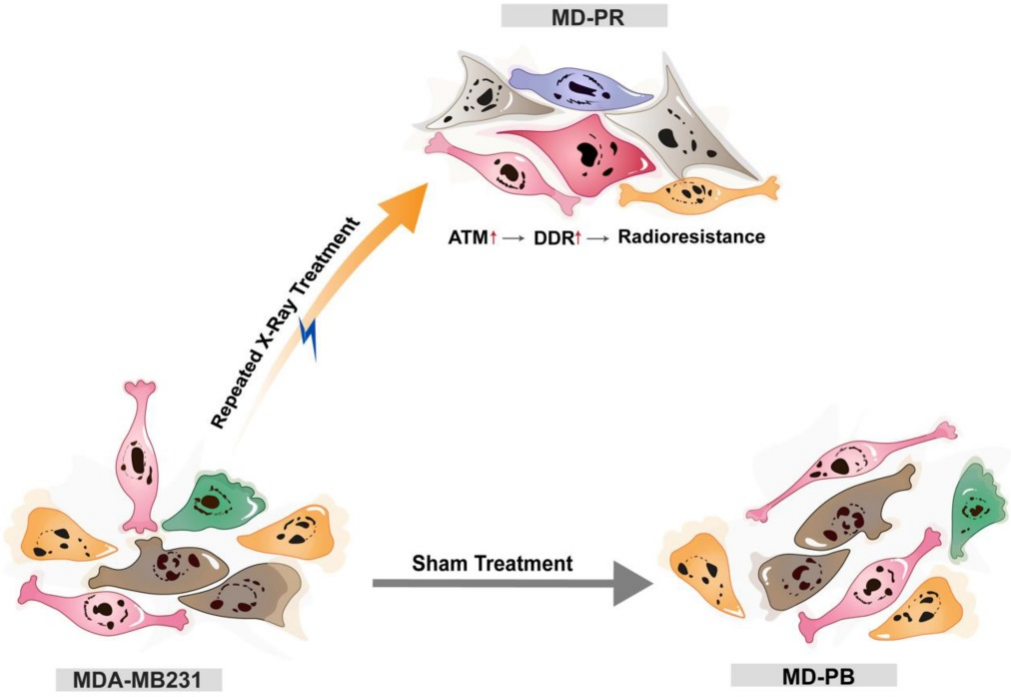
** The model of the possible mechanism of radioresistance.** Breast cancer cell line MDA-MB231 cells were either sham-treated (MD-PB) or repeated treated with X-ray radiation (MD-PR). In MD-PR cells, the elevation of ATM expression level leads to increased DNA damage repair (DDR) efficiency, which ultimately caused the enhanced radioresistance in MD-PR cells compared with MD-PB cells.

Together, these findings led us to propose that cancers cell may adapt to radiation and develop resistance to irradiation through increasing the expression of ATM, which could accelerate DSBs repair efficiency and reduce radiation-induced cell apoptosis (Fig. 6). Nonetheless, the elevation of ATM just explained part of the reason for enhanced radioresistance. After inhibition of ATM, through specific inhibitor or siRNA, MD-PR cells were still more radioresistant compared with MD-PB cells (Fig. 4B, 5B), suggesting that some other pathways also contribute to the acquired radio-resistance in MD-PR cell line. Of note, compared with ATM inhibitor, the application of siRNAs against ATM further narrowed the gap of radiosensitivity difference between MD-PR and MD-PB. It could be explained by residual ATM kinase activity (Fig. 4A, C) or other functions of ATM independent of its kinase activity 35.

In our experiments, γ-H2AX kinetic was used as the measurement of DSB repair efficiency because higher γ-H2AX expression correlates with more unrepaired DSBs 11. MD-PR cells presented earlier diminishment of γ-H2AX compared with MD-PB, which was in accordance with increased NHEJ repair efficiency. While knock-down of ATM using siRNA abrogated γ-H2AX formation at an early time after irradiation (Fig. 5C), inhibition of ATM with specific inhibitor delayed initial γ-H2AX phosphorylation in both cell lines to around 4 hours post-irradiation (Fig. 4F). This may be explained by the different treating time between these two experiments. CP466722 was added 2 hours before irradiation and removed 3 hours later, which may cause an increase of γ-H2AX at 4-hour post-irradiation. siRNAs were transfected 24 hours before cells were reseeded, while the last sample was collected at 72-hour post siRNA transfection, when the effect of the knockdown was still obvious (Fig. 5A). The knockdown of ATM produced detectable residual phosphorylation at the late time point (8-hour, Fig. 5D), which suggested an inhibition of late steps in the DSB process 36.

In previous clinical studies, ATM inhibitors have been proved effective in sensitizing tumor cells to radiation and many trials are ongoing 21, 37. Our findings further support the strategy of targeting ATM alone or in combination with other therapeutics for personized therapy 38, 39. ATM is thought to be the core protein for HR 31. However, elevated expression of ATM did not produce an increased efficiency of HR in our experiments. It is evidenced elsewhere that, ATM is not essential for HR and that HR-driven processes could take place in its absence 31, 40. Also, considering HR is strictly restricted to G2 and S phase and the cell cycle distribution had no difference between MD-PB and MD-PR cells, the unchanged HR efficiency is reasonable.

It remains unknown how ATM expression is upregulated in MD-PR cells after repeated radiation treatment. Neither is the mechanism of ATM-mediated increased DNA repair efficiency. It is possible that even after a long time since the last radiation treatment, the adaptive effect 41 still exists in MD-PR cells and prepares them for a new even larger dose of radiation and ATM is part of the adaptive response. Future work should focus on ATM conjugated proteins, especially those that can potentially affect DNA damage repair pathways, to better reveal the relation between ATM and increased DDR efficiency in radioresistance development.

## Conclusion

In our present work, we established a relatively radio-resistant breast cancer cell line and proposed an explanation for the emergence of radioresistance in cancer cells and the failure of radiation therapy. When cancer cells receive radiation damage, the repair efficiency of DSBs is upregulated, thus avoiding the fate of apoptosis and necrosis and decreasing radiation sensitivity of cancer. The enhanced repair efficiency might be mediated by elevated expression of ATM and its activated form p-ATM (ser1981). To our knowledge, this is the first evidence correlating elevated DSB repair efficiency with therapy-induced radioresistance.

## Supplementary Material

Supplementary figures and table.

## Abbreviations

DSBs: double-strand breaks
DDR: DNA damage repair
HR: homologous recombination
NHEJ: non-homologous end joining
ATM: Ataxia telangiectasia mutated
