# Supplementary material for: ATM Expression Is Elevated in Established Radiation-Resistant Breast Cancer Cells and Improves DNA Repair Efficiency
Source: Int J Biol Sci. 2020 Feb 4;16(7):1096–106. doi: 10.7150/ijbs.41246 (PMC7053315; doi:10.7150/ijbs.41246)
Supplement: Supplementary file 1 — Supplementary figures and table. [file ijbsv16p1096s1.pdf]

Lei Bian et al`s Supplementary Materials

Fig. S1

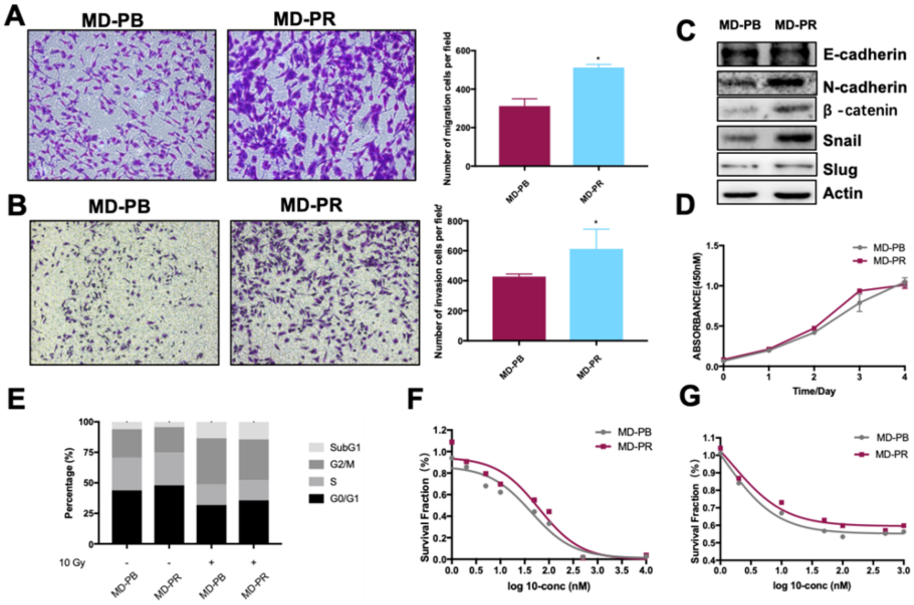

Fig. S1. Functional assays of MD-PB and MD-PR cells. (A, B) Representative pictures of cell migration assays (A, left) and invasion assays (B, left) were taken under microscope of 200X magnification. Cells in at least 5 fields were calculated and results of at least 3 independent experiments were used for statistical analysis (A, B, right). (C) EMT markers were detected in MD-PB and MD-PR cells by western blotting. (D) Cells were seeded in 96-well plate and tested using CCK-8 kit at indicated time for analysis of proliferation rate. (E) Cells were ionized with or without 10Gy irradiation and then incubated for 48 hours before submitted to cell cycle analysis. For analysis of sensitivity to different chemotherapeutics (F, G), cells were seeded, 24 hours later, Doxorubicin (F) and Paclitaxel (G) at different concentrations or DMSO as control were added to cell culture medium. 48 hours later, absorbance at 450nm was detected using CCK-8 kit. Surviving fraction was calculated as (Absorbance at certain concentration group)/ (Absorbance at DMSO group).

Fig. S2

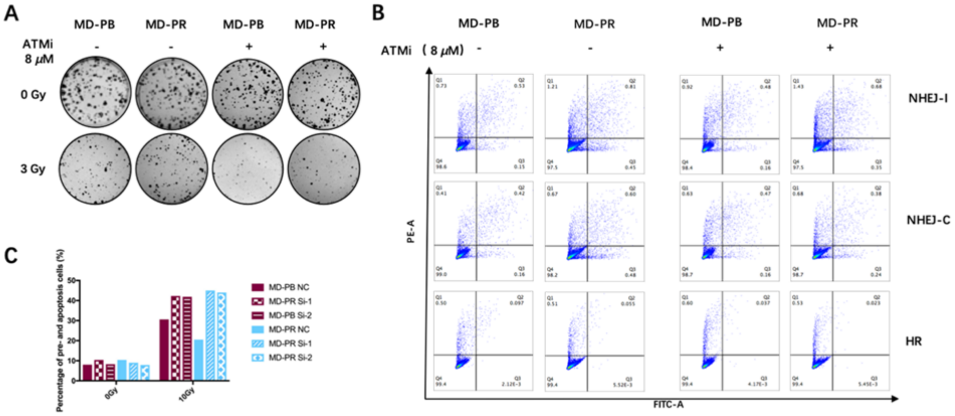

Fig.S2. Colony formation assays (A), DNA damage repair efficiency (B) and apoptosis analysis(C) of MB-PB and MD-PR cells under different condition. (A) Cells were seeded and pretreated with ATMi or DMSO 2 hours before receiving 0 or 3Gy of irradiation. Cells were then incubated 3 hours before medium were completed changed for fresh medium. 2 weeks later, colonies were fixed and stained. Representative pictures were shown. (B) Flow cytometry results of the impact of ATMi inhibition on NHEJ and HR repair efficiency. Methods were described in preceding part of the article. Representative results of flow cytometry analysis results were shown. (C) As describe in preceding text, cells were transfected, irradiated at 24 hours later and subjected to apoptosis analysis 48-hour post 0 or 10 Gy of X-ray. Percentage of apoptotic cells were calculated as before.

Table. S1. List of antibodies used in the text.

Product	Product Name	Product Company	Product Number
Primary antibody	Anti-β-actin	Transgene	HC-201
	Anti-Phosphor-Histone H2A.X (Ser139)	Santa Cruz	sc-517348
	Anti-Snail	ABclonal	A5243
	Anti-E-Cadherin	CST	#3195
	Anti-N-Cadherin	CST	#13116
	Anti-Slug	CST	#9585
	Anti-β-Catenin	CST	#8480
	Anti-ATM	CST	#2873
	Anti-phosphor-ATM	CST	#5883
	Anti-MRE11	CST	#4847
	Anti-RAD50	CST	#3427
	Anti-DNA-PKcs	CST	#4602
	Anti-KU80	CST	#2180
Secondary antibody	Anti-rabbit IgG, HRP linked	CST	#7074
	Anti-mouse IgG, HRP linked	CST	#7076
	Donkey anti-mouse IgG Alexa Fluor conjugate	Life Technologies	#A-21201
